# 孤立髓系肉瘤

**DOI:** 10.3760/cma.j.issn.0253-2727.2021.10.015

**Published:** 2021-10

**Authors:** 婧 王, 晓军 黄

**Affiliations:** 北京大学人民医院、北京大学血液病研究所 100044 Peking University People's Hospital, Peking University Institute of Hematology, Beijing 100044, China

孤立髓系肉瘤（Isolated myeloid sarcoma，IMS）是指不成熟髓系前体细胞在骨髓外形成的实体性肿瘤，骨髓未累及，且没有急性髓系白血病（AML）、骨髓增生异常综合征（MDS）或骨髓增殖性肿瘤（MPN）病史。由Burns[1]在1811年首次报道，1966年Rappaport[2]重新命名为粒细胞肉瘤，1988年Davey提出髓外髓细胞肿瘤的概念，IMS的概念及命名在WHO 2008年颁布的髓系肿瘤的新分类中被正式采纳[3]。IMS的文献报道多以个例报道为主，虽然病例数量大的报道较少，且多为回顾性的总结分析，缺少前瞻性的临床试验研究，但关于影响IMS预后的因素，尤其是IMS缓解后的治疗策略的研究仍有一些进展，而且是目前临床医师尤其关注的问题。

一、临床特点及预后

1. 发病率：髓系肉瘤（MS）在临床上可以是原发孤立的IMS，也可以同时伴随外周血及骨髓累及，可以表现为缓解的AML的髓外复发，也可以表现为是MDS、MPN或MDS/MPN的病情进展。MS男性稍多于女性，比例约为1.2∶1，中位年龄56岁（1个月～89岁）。MS是比较少见的恶性血液系统肿瘤，成人总发病率2/100万，儿童发病率0.7/100万，占AML的1.4％～9％，IMS占AML的1％～2％，IMS若不积极治疗，在5～12个月内有进展为AML的风险，进展后生存期短[3]–[7]。

2. 发病部位：IMS可发生于全身各器官系统，如皮肤、淋巴结、骨、骨膜、软组织、神经系统、消化系统、乳腺等部位，表现为实体肿块，类似实体瘤，小于10％表现为多部位。多数患者以局部肿块形成或肿块引起的局部压迫症状为首发表现。Yui等[8]报道96例MS，累及部位：皮肤软组织37例（38％）,淋巴系统17例（18％）,胃肠道泌尿生殖系统14例（15％）,神经系统9例（9％），乳腺3例（3％），多部位或其他16例（17 ％）。Goyal等[9]近期分析94 000例AML患者，746例（0.8％）诊断MS，最常见的累及部位分别为：软组织31.3％，皮肤/乳腺12.3％，消化系统10.3％。波兰多中心分析39例MS，累及部位依次为中枢神经系统（18％）、软组织（8％）、淋巴结（6％）、皮肤（6％）、骨（5％）、胸膜（4％），21例（54％）累及多部位[10]。Claerhout等[11]分析72例MS患者（IMS或继发MS），结果提示累及部位与只是IMS或曾诊断AML无关。

3. 免疫表型与细胞遗传学：免疫组织化学染色是诊断IMS最为有效且简单易行的方法，可根据细胞表型来判断IMS的类型及细胞分化程度，IMS绝大部分表达髓系相关抗原，CD68表达最常见，MPO阳性率达77％～97％，CD43阳性率可达100％，其次可表达CD117、CD15、CD13、CD33、CD99等。55％的患者伴有细胞遗传学及分子学异常，包括t（8;21）、inv（ 16）、单体7、+8、+4、单体16、16q−、5q−、20q−、MLL基因重排、NPM1^+^、FLT3-ITD^+^等[3],[7]。

4. 预后影响因素：细胞遗传学与预后相关性正在引起重视。Yui等[8]报道96例MS患者，中位年龄53（17～83）岁，男性64例（67％），原发64％（61例），继发36％（35例，血液肿瘤或治疗相关），74例进行了细胞遗传学分层：预后好7例（9％），预后中等45例（61％），预后差22例（30％）。中位随访135周，57例（59％）死亡。49例（70％）获得完全缓解（CR），26例（53％）复发。细胞遗传学分层好、中、差组OS时间分别为169、52和17.5个月（*P*＝0.040）。有无骨髓累及组中位OS时间分别为17和20个月（*P*＝0.400）。原发及继发于AML的MS中位OS时间分别为52和11.5个月（*P*<0.001）。结果提示细胞遗传学好及原发MS与预后好相关。Stein-Wexler等[12]报道t（8；21）（q22；q22）是良好预后指标；Byrd等[13]曾报道伴t（8；21）（q22；q22）染色体改变的AML患者，出现髓外MS者比无髓外侵犯者缓解率低，生存期短。

有研究发现治疗方法的选择与患者的预后有一定的相关性。Yamauchi等[14]发现如果单纯使用放疗或手术治疗等局部治疗，IMS患者在3～6个月出现骨髓受累，而接受全身系统化疗的患者转化为AML时间可延长至12个月。杨玲等[15]报道1例MS患者接受局部治疗，8例MS患者接受系统化疗。接受局部治疗的l例患者在病程第6个月出现骨髓受累，进行化疗的8例患者中5例在中位病程14（2～34）个月转化为AML。提示全身系统化疗有助于延长MS的AML转化时间。另外有研究报道缓解后异基因造血干细胞移植（allo-HSCT）可以延长IMS患者生存[6],[16]。

目前大多数研究者认为临床表现及治疗反应不受年龄、性别、累及部位、AML/MDS/MPN病史、组化表现、免疫分型等因素影响。

二、治疗

由于IMS发病率低、缺乏前瞻性研究，目前还没有统一的治疗建议。IMS的治疗与以下因素有关：IMS的部位（皮肤、中枢神经系统或其他）、IMS发生的时间（首发、化疗后复发、移植后复发）、患者的年龄、患者的一般状况等。治疗选择包括：化疗、放疗、移植、靶向治疗及免疫治疗[17]–[19]。

1. IMS是否需要治疗：Neiman等[20]曾报道在15例未予化疗的IMS患者中，13例（87％）在中位10.5个月后进展为AML。Yamauchi等[14]对72例IMS患者不同疗法的疗效进行了比较，32例予手术切除或放疗而未进行全身化疗的患者中，28例（88％）在3～6个月出现骨髓受累，11个月内进展为AML；而42例予全身化疗的患者中，25例（58％）在随访11个月后仍无骨髓浸润表现，提示全身化疗比手术切除及放疗更能延长无骨髓浸润期，延缓向AML的进展。鉴于几乎所有IMS患者最终会进展为AML，IMS需要治疗已达成共识。

2. IMS如何治疗：

（1）诱导治疗：诱导治疗的效果在多个研究中报道。Tsimberidou等[21]报道23例IMS患者采用阿糖胞苷、去甲氧柔红霉素及氟达拉滨诱导化疗，CR率达69％。在一项回顾性研究中，9例IMS患者采用阿糖胞苷/蒽环类药物为基础的化疗，CR率为71％[22]。由于IMS领域治疗比较的资料有限，最优的治疗方案并不明确，但是阿糖胞苷为基础的治疗模式是最常用的，目前，对于IMS的诱导治疗建议AML样化疗作为标准治疗[23]–[24]。

（2）缓解后治疗：IMS缓解后治疗经验非常有限，是否所有患者需要缓解后治疗目前没有前瞻性循证证据支持。一般根据患者的年龄、患病部位、MS的范围、患者一般状况、细胞遗传学及分子学异常的情况综合决定[18]。

缓解后是否行造血干细胞移植（HSCT）虽然没有前瞻性研究作为循证学建议，但正逐渐在引起重视。Pileri等[16]的研究结果显示IMS患者在CR_1_状态下行HSCT明显改善OS，移植组和未移植组48个月OS率分别为76％和0（*P*<0.001）。Chevallier等[25]报道99例患者（30例IMS，69例AML伴MS）行HSCT，5年OS和无白血病生存（LFS）率分别为48％和36％，在多因素分析中，不良细胞遗传学异常明显缩短LFS时间，移植前IMS处于CR状态明显提升LFS。Antic等[6]分析了12例IMS患者的治疗。男性8例，中位年龄39岁，化疗7例（3例联合HSCT），手术5例，随访期内，9例患者死亡，中位存活13个月，无事件生存（EFS）时间8个月，OS与初始治疗方式无明显相关性，但化疗与HSCT分别是提高EFS率的独立预后因素，化疗联合allo-HSCT可以明显延长EFS时间，<40岁联合化疗/allo-HSCT明显影响EFS。汪清源等[26]分析15例IMS患者，中位年龄44（13～52）岁，发病部位累及乳腺、子宫、胃肠道等，中位生存时间30.5（4～100）个月，AML化疗方案及早期CR状态下行allo-HSCT可改善OS。因此，allo-HSCT对于IMS很有可能成为一个潜在有效的缓解后治疗选择。

放射治疗的作用相关研究资料有限且并结论不统一。Tsimberidou等[27]报道认为化疗基础上联合放疗改善生存；Feller等[28]的一项回顾性分析71例患者的研究结果显示化疗基础上联合放疗并没有使患者获益更多。目前对于IMS化疗疗效不佳者建议加用放疗，如果放疗毒性可耐受，也可以作为巩固治疗的选择[28]，对于移植后出现IMS复发的患者也建议给予放疗[24]。

大多数靶向治疗的临床试验是不包括IMS的，这些潜在的靶向基因在IMS中的预后意义目前还没有相应的数据支持。

对于不适合强化疗的患者，可以采用小剂量治疗，放疗和手术也可以用于改善症状。对于老年和（或）不适合强化疗的IMS患者，去甲基化药物也有成功的报道[30]–[31]。

（3）复发后治疗：IMS复发很少见，一般都是骨髓复发的先兆，从IMS复发至骨髓复发中位时间为7（1～19）个月[32]，即使是局部复发，需要全身系统治疗而不是只针对局部治疗[24]。化疗后复发的患者没有标准的挽救方案，可以采用之前没有用过的AML挽救治疗方案再诱导，之后建议行allo-HSCT，对于化疗疗效不充分患者可选择放疗辅助[17],[24]，移植后IMS复发也是全面复发的先兆，预后极差，中位生存时间4个月[33]，根据患者一般状况可以选择供者淋巴细胞输注、化疗、二次allo-HSCT、甲基化治疗及靶向治疗等[17],[24],[34]。

总之，对于IMS，首先建议进行全身系统治疗,诱导治疗可采用AML样诱导方案，诱导治疗后疗效不佳者建议加用放疗，诱导缓解后有条件者建议行allo-HSCT，老年及不适宜强化疗患者可采用去甲基化治疗，随着二代测序的普及，靶向治疗有可能成为未来治疗选择。

鉴于IMS的发生率低，所有治疗建议都需要未来前瞻性随机临床研究进一步验证。
